# Developing the Next Generation of Nursing Disciplinary Leaders in Higher Education: Protocol for a Sequential Mixed Methods Study

**DOI:** 10.2196/40677

**Published:** 2023-03-16

**Authors:** Melissa Slattery, Carol Grech, Rachael Vernon

**Affiliations:** 1 Department of Clinical and Health Sciences University of South Australia Adelaide Australia

**Keywords:** academic, attributes, behaviors, characteristics, higher education, leadership, nursing, qualities

## Abstract

**Background:**

Leading nurse education and research in the higher education (HE) sector has become increasingly challenging over the last decade with many universities in Australia and New Zealand having undergone academic restructuring. The future of HE faces many challenges including recruitment of suitably qualified staff to lead teaching and research and advance professional disciplines. Increasing cultural diversity of the Nursing workforce and the communities’ nurses serve, and the identification of cultural attributes in the context of racial inequities exposed by the pandemic and the climate emergency suggest different forms of leadership may be required in the future by those leading nurse education in the HE sector. Currently, there is a dearth of research evidence that identifies the qualities, behaviors, and characteristics (collectively identified as core attributes) required by nurse academic leaders.

**Objective:**

This research aims to identify an evidenced based set of core attributes that are required to lead the discipline of Nursing in the Australian and New Zealand HE sectors.

**Methods:**

This research is using a 2-phase sequential mixed methods design incorporating a scoping review; and Delphi technique. In phase 1, a scoping review will be undertaken to identify the qualities, behaviors, and characteristics that can influence the evolution of the next generation of academic nurse leaders. A set of draft statements and questions will be prepared based on analysis of findings from the review. Phase 2 uses Delphi technique consisting of e-survey rounds with experts in leading nursing faculties in Australia and New Zealand. An Expert Advisory Group will consider the initial set of draft statements and questions from phase 1. Consistent with Delphi technique, a series of “rounds” will then occur using an e-survey method. Established leaders (Professors and Associate Professors who are members of the Council of Deans Australia and New Zealand) will rate their level of agreement to statements on the qualities, behaviors, and characteristics required to lead the discipline of nursing in the HE sector in Australia and New Zealand.

**Results:**

The findings of the scoping review will identify what is currently known about the qualities, behaviors, and characteristics of academic nurse leaders. Quantitative and qualitative results from the Delphi study will initially be reported in separate manuscripts for publication. It is projected that a final paper will be prepared from aggregated research data and outline how the findings can inform the preparation of future academic nurse leaders.

**Conclusions:**

The generation of an evidenced-based set of core attributes will serve to inform the next generation of academic nurse leaders including informing recruitment processes and postgraduate nurse leadership programs. It is anticipated that the data sets and findings will be transferrable to other disciplines within HE to aid in future-proofing discipline-based expertise and leadership in the context of academic restructure.

**International Registered Report Identifier (IRRID):**

PRR1-10.2196/40677

## Introduction

### Background

Nurse leaders exist across the professional spectrum including clinical practice, education, management, policy, and research. Nursing is now a well-established discipline in higher education (HE) in Australia and New Zealand, creating knowledge through research inquiry and producing graduates for employment in health through accredited program pathways. However, there are now significant challenges in HE to growing the next generation of academic nurse disciplinary leads [1]. A key focus of the nursing profession remains the commitment to teaching, supervision of students, and assessment to develop the nursing workforce across the professional spectrum [2]. Where once academic appointments in HE were seen as an attractive option for RNs seeking to move away from clinical practice to a well-remunerated workplace setting with autonomy and flexible working arrangements, this is no longer the case. As an applied profession, there is the expectation that registered nurses **(**RNs) working in HE have significant years of clinical experience to effectively mentor students and provide the next generation of nurses with foundation knowledge on which they can safely commence their careers in industry [3]. Despite these high expectations, there is a disparity between health industry salaries that sit significantly higher than a commensurate salary of a Lecturer (or level A and B academic). In contrast, a career in HE requires a postgraduate qualification and for advancement, doctoral preparation is often an essential criterion. As an applied discipline, nurses employed in the HE sector to teach nursing theory and practice in Australia are required to be an RN and hold a qualification higher than the program level that they are teaching into or have equivalent professional experience [4]. These requirements are embedded in Standard 3.14 of the current Registered Nurse Accreditation Standards 2019 through the Australian Nursing and Midwifery Accreditation Council on behalf of the Nursing and Midwifery Board of Australia. In New Zealand, individuals employed as a nurse academic must be an RN and hold a relevant master’s degree (or have a professional development plan in place to obtain a master’s degree within 4 years of initial appointment), have completed a program in adult teaching and learning within 2 years of appointment, and be involved in research and scholarship activities. These requirements are embedded in Standard 3.10 of the current RN Education Programme Standards (2021) [5]. Commitment to gaining the requisite qualifications for a career in HE can present as a financial burden and requires a considerable time commitment. For these reasons, RNs with the potential to be excellent academic nurse leaders may not find a move to HE as an attractive alternative to staying in clinical practice.

COVID-19–induced cost-saving measures in HE have compounded the need for fiscal restraint measures and have placed additional pressures on university staffing budgets [6]. While now in a recovery phase staff recruitment is a growing concern across the sector raising questions as to how the next generation of nurse academics and leaders in HE will be supported into a sustainable career pathway.

The HE sector in Australia and New Zealand is at a crossroad. As a key contributor to the Australian and New Zealand economies, the HE sector holds a significant role in ensuring workforce supply needs are met and graduates meet diverse employability requirements. The past 15 years have seen a change in the global economy resulting in streamlining of service delivery to reduce operational costs and a stronger dependence of local society on the income brought about through education. This altering climate has been at the forefront of structural changes across Australian and New Zealand HE. The key considerations of HE in the 21st century include internationalization, student learning outcomes, diversification, racial inequities and gender equity, societal need, and strategic planning [7-11]. There are also challenges for leaders going forward in the context of racial inequities shown during the pandemic and climate emergency. Leaders into the future may need to demonstrate different forms of leadership and have additional skill sets that recognize these significant challenges.

For many RNs working within the HE sector, career advancement is another challenge. Structural challenges and changes have become increasingly common, with some nursing schools losing standalone “disciplinary” status as they become part of larger multidisciplinary cognate units led by nonnurses. With such significant structural changes there has been an increase in the number of fixed-term (nontenured) teaching specialist positions; an increased reliance on a casualized workforce; and in many instances fewer senior nurse academic leadership roles, or these roles have cross-disciplinary implications.

### Aim

This research aims to identify the qualities, behaviors, and characteristics that exist within academic nurse leaders (senior, established leaders) to provide the evidence in which to develop a set of core attributes to support the development of emerging leaders. Core attributes comprise the key qualities, behaviors, and characteristics that exemplify the essence and culture academic staff should emulate [12].

### Objectives

This research will identify the qualities, behaviors, and characteristics (core attributes) that are exhibited by exceptional nurse leaders who are associate professors or professors (levels D and E) in Australian and New Zealand HE schools and faculties of nursing. These concepts will be explored as the foundation to developing a successful career pathway for emerging academic nurse leaders. This research is using a 2-phase sequential mixed methods design incorporating a scoping review and the Delphi method. In phase 1, a scoping review will be undertaken to identify the qualities, behaviors, and characteristics that can influence the evolution of the next generation of academic nurse leaders. Phase 2 uses the Delphi technique consisting of e-survey rounds with experts in leading nursing faculties in Australia and New Zealand. An Expert Advisory Group consisting of past or current Deans or Heads of nursing faculties will consider the initial set of draft statements and questions arising from the analysis of the scoping review findings. Consistent with the Delphi technique, a series of “rounds” will then occur using the e-survey method. Established leaders (professors and associate professors who are members of the Council of Deans of Nursing and Midwifery [Australia & New Zealand]) will rate their level of agreement via a Likert scale to statements on the qualities, behaviors, and characteristics required to lead the discipline of nursing in the HE sector in Australia and New Zealand. The questions for each round will be based on the findings of the previous round until consensus (set at 90% agreement) has been achieved. Participants will also have the opportunity in the e-survey to provide qualitative comments.

### Primary Research Question

What are the core attributes (qualities, behaviors, and characteristics) required to succeed in the role of academic nurse disciplinary lead?

## Methods

### Study Design

#### Overview

Mackenzie and Knipe [13] argued the existence of a “pragmatic paradigm” that is not committed to a particular view of the world. Pragmatist researchers focus on the “what” and “how” of the research problem [14]. Pragmatism is seen as the paradigm that provides the underlying philosophical framework for mixed methods research [15,16]. The pragmatic paradigm places the research problem as central and applies all approaches (methods) to understanding the problem [14]. With the research question “central,” data collection and analysis methods are chosen as those most likely to provide insights into the question with no philosophical loyalty to any alternative paradigm [13]. To achieve the research objectives, the research design must capture the qualities, behaviors, and characteristics that are required to succeed in the role of academic nurse leader (qualitative data) and must also capture the degree to which established senior academic nurse leaders and the Expert Advisory Group believe these are agreeable (quantitative data). This is to ensure that the data captured meet the expectations of those who make employment-related decisions (ie, senior leaders) and resonate with the emerging leaders who are looking to pursue a career pathway in nurse academe. A sequential mixed methods approach with 2 clearly defined methods has been chosen as the research will be an iterative process, specifically with the scoping review findings informing the Delphi method [17]. This approach requires flexibility and adaptability while maintaining the research question as central to the design.

#### Phase 1: Scoping Review

A scoping review of the literature will be undertaken to identify qualities, behaviors, and characteristics that are considered desirable in academic nurse leaders. A scoping review method has been chosen as it enables the mapping of a wide-ranging set of relevant literature and key concepts underpinning the research topic [18]. While an initial review of published research showed a gap in this area, there were surmountable findings to support a search of the literature that specifically targeted the qualities, behaviors, and characteristics that support career success. This approach allowed for consideration of published and gray literature and the identification of a range of potential qualities, behaviors, and characteristics and the development of an early set of core attributes that will be presented to an Expert Advisory Group (phase 2, round 1). The scoping review aligns to the Preferred Reporting Items for Systematic Reviews and Meta-Analysis Extension for Scoping Reviews (PRISMA-ScR) published in 2015 by Peters et al [19]. Specific questions pursued during the scoping review included: What are the qualities, behaviors, and characteristics required for a nurse disciplinary lead? What are the qualifications required to work as a nurse disciplinary lead? What are the intrinsic and extrinsic factors that may affect an individual’s ability to succeed as an academic nurse leader? Does mentorship have a positive impact on academic nurse leadership skill development? What type of framework could assist supporting nurses into the role of academic nurse leader? Searches for MEDLINE, Embase, Emcare, ProQuest Dissertations and Theses Global, and Open Access Theses and Dissertations were undertaken. An extraction table was created to identify and analyze how each article addressed one or more of the research questions and a content analysis was conducted [20]. The findings of this review will be written up as a qualitative data set informing an early list of qualities, behaviors, and characteristics that will be built on and refined through phase 2.

#### Phase 2: Delphi Method

A Delphi method will be conducted to establish consensus on the core attributes required for future academic nurse leaders. The Delphi technique has been chosen due to the ability to refine the thoughts and ideas of a large cohort of experts to form a consensus view, defined at ≥90% agreement on a set of core attributes [17,21,22]. While agreement of ≥80% is deemed sufficient to validate content, the researchers have set an agreement of ≥90% to ensure a high level of validity [22].

#### Round 1: Expert Advisory Group

In round 1 of the Delphi method, an Expert Advisory Group will be sought to confirm the broader understanding and application of the preliminary list of key qualities, behaviors, and characteristics for academic nurse leader success as informed by the scoping review in phase 1. The research will specifically target senior leaders from Schools of nursing within Table A or public universities throughout Australia and New Zealand [23]. These Table A or public universities have been chosen due to their strong research divisions, participation in the development of nursing academe (through the Council of Deans of Nursing and Midwifery [CDNM; Australia & New Zealand]), and their support of “balanced” academic positions—where individuals are required to attend to both teaching, research, and professional engagement duties to maintain their academic skill sets. Senior leaders have been identified to participate in this Expert Advisory Group due to their significant experience within academe, their influence and insight in developing staff, and the distinctive understanding and views they hold of academic hierarchy having succeeded in the role of associate professor or professor. Analysis of these traits, followed by further testing with established leaders, will assist in the creation of a set of agreed core attributes to support succession planning and growing the next generation of academic nurse disciplinary leads. Such measures are necessary to address a potential future shortage of transformational nurse leaders who are able to influence future academic leadership roles in HE during a time where many universities are undergoing restructure. Disciplinary leads are the leaders who hold decision-making, executive roles within Table A or public universities and are responsible for leading the Discipline [24]. Participants will be invited to an internet-based meeting and asked to review the list of qualities, behaviors, and characteristics determined in phase 1, identify definitions for terms with multiple meaning, identify gaps, and address these gaps. As an iterative process, the data will then lead to the undertaking of round 2.

#### Rounds 2 and 3: Delphi e-Survey

In round 2 of the Delphi method, the refined data from round 1 will be tested through at least 2 rounds of the Delphi e-survey targeting all established leaders. The Delphi e-survey has been chosen to facilitate the collation of information beyond the Expert Advisory Group as it facilitates the easy circulation of survey material and collation of high-quality data across Australia and New Zealand at the convenience of the research participant (ie, no restraint due to time zones or travel required) [21]. The Delphi e-survey will aim to further refine any areas of ambiguity in the list of qualities, behaviors, and characteristics while also gauging prioritization to inform the development of a final list of core attributes. Content analysis will be undertaken after round 2. In round 3, the findings of round 2 will be presented to the participants of round 2. Participants’ reactions to the findings will be sought. Consensus on the list of core attributes will be defined as ≥90% agreement [22,25]. The number of rounds of the Delphi method will be dependent on the establishment of consensus [25].

#### Round 4: Achieving Consensus

In round 4, the Expert Advisory Group will test the consensus opinion of the established leaders. The purpose of testing this research with the Expert Advisory Group is to ensure the proposed attributes and steps toward the research objectives meet the practical requirements of the role and end user—the future academic nurse disciplinary leads. As round 4 of the research will be an iterative process, the number of rounds will be dependent on the establishment of consensus and, if needed, further refine the core attributes. Content analysis will be used to further validate if consensus has been met across the participants in terms of the accuracy and resonance of the final list of core attributes and any further areas for considerations or areas for investigation. Consensus on the final list of core attributes will be defined as ≥90% agreement [22,25].

### Mixed Methods Approach

The qualitative data captured throughout the Delphi method will be synthesized through content analysis. Content analysis is advantageous in this research design as it allows for the analysis of qualitative data while also quantifying its importance [20]. Content analysis is suitable to provide simple reporting of common issues identified in the data, which will likely be necessary to refine the qualities, behaviors, and characteristics into a list of core attributes for consideration in round 2 of the Delphi method. Content analysis allows for coding of the data to assume a quantitative measure—or weight—to which the qualities, behaviors, and characteristics can be measured in degree of importance or value [26,27].

### Setting

It is noted that in some instances, schools or faculties of nursing in Australia include Midwifery to provide a multidisciplinary school reflective of a single regulatory board for both professions [28]. Universities in New Zealand do not have this “merged” model as Nursing and Midwifery have separate regulatory authorities, that is, the Nursing Council and the Midwifery Council [29,30]. This research will focus on the discipline of nursing. Although some nurse leaders may also be registered as a Midwife, midwifery has been excluded from this research to maintain comparable data specific to nurse leadership within academe. In examining qualities, behaviors, and characteristics exclusively within the discipline of nursing, this study will take on a similar approach to the research conducted by Carryer et al [31] in 2007, which explored the core role of nurse practitioners specifically within a clinical setting in Australia and New Zealand. Carryer and colleagues’ study [31] recruited participants through the Nurses Board or Council of each respective country and excluded midwifery. A similar approach was again taken by Gardner and colleagues [32] in 2016. In this research, specific steps will be taken to ensure trans-Tasman distribution of participation through improving accessibility (eg, using ZOOM) and consideration of terminology to target both Australian and New Zealand HE sectors’ requirements.

### Participants

#### Overview

This study will focus on the recruitment of a sample population from within schools and faculties of nursing at identified Table A or public universities in Australia and New Zealand. These public universities have been chosen due to their strong research divisions, delivery of education in the nursing discipline, participation in the development of nursing academe (through the CDNM), and their support of “balanced” academic positions—where individuals are required to attend to both teaching and research duties to maintain their academic skill sets. Thirty-three Australian universities and 5 New Zealand universities have been identified—although it is noted that there have been significant changes in discipline delivery in some universities over the past 2 years and this may impact inclusion at the time of phase 2.

Participation for rounds 1 and 4 (Expert Advisory Group) will target senior leaders. The commitment of a participant to the Expert Advisory Group is likely to reflect their interest and involvement with the issue being addressed [22]. Recruitment of this Expert Advisory Group will be through direct invitation, initially made via email (through the CDNM) and then followed up by telephone. Representatives from Australia and New Zealand will be chosen because of the similarities in the standards of practice and regulatory structures for nursing.

#### Inclusion Criteria

The inclusion criteria are as follows: (1) well-renowned and recognized senior leaders who have currently or previously represented their institution as the named Member of the CDNM (Australia and New Zealand); (2) currently or previously employed as a dean, head of school, or head of discipline; and (3) actively working within Table A or public universities in Australia and New Zealand.

#### Exclusion Criteria

The exclusion criteria are as follows: (1) designated proxies to the CDNM (Australia and New Zealand) and (2) members of the PhD candidate’s supervisory panel who would meet the inclusion criteria.

#### Recruitment

##### Overview

A minimum threshold of 5 participants has been set for the Expert Advisory Group to establish initial priorities. Prospective participants will be recruited through email contact from the researcher, outlining the purpose of the research, participation requirements, and consent. If available, the researcher will also approach the CDNM for their support in promoting the research initiative to improve the likelihood of participation.

Participation for rounds 2 and 3 (Delphi e-survey) will target established leaders.

##### Inclusion Criteria

The inclusion criteria are as follows: (1) established leader within the discipline of nursing, including those responsible for leading nursing programs; (2) employed as an associate professor (level D or E); (3) actively working within Table A or public universities in Australia and New Zealand; (4) is an active member of the CDNM (Australia and New Zealand); and (5) is a designated proxy to the CDNM (Australia and New Zealand).

##### Exclusion Criteria

The exclusion criterion is not participating in CDNM (Australia and New Zealand).

#### Recruitment: Rounds 2 and 3

A minimum threshold of 15 participants has been set for rounds 2 and 3 of the Delphi method to refine and test the resonance of the qualities, behaviors, and characteristics to create the draft list of core attributes. Prospective participants will be recruited through similar means to round 1 (direct email contact through the CDNM); however, they may also be recruited through referral from round 1 participants or through advertising, to ensure a broad capture of evidence.

### Ethical Considerations

#### Approval

Ethics approval was obtained for all phases of this study from the University of South Australia’s Human Research Ethics Committee (HREC) on April 30, 2020 (202862). Phase 1 of the research was a scoping review and as a literature-based form of inquiry it did not require ethics approval; however, the researchers noted that there are still some considerations regarding scoping review methods that include studies wherein the researchers have included human participants without approval of a duly constituted ethics committee. Key ethical considerations for phase 2 of this research are obtaining informed consent, ensuring that participants’ details are treated in a manner that ensures an appropriate degree of privacy, and the safe storage of confidential data collected within the research.

#### Consent

Participants in phase 2 of the research will be provided a Participant Information Sheet (PIS) specific to the phase of research. The PIS details the purpose of the research including the aim, objectives, and potential outcomes. The PIS provides prospective participants with details on participation, including what participation would involve, potential benefits, potential risks, confidentiality, and strategy for managing any adverse events. If prospective participants have any unanswered questions, the contact information of the research team is provided within the PIS, thereby further enabling informed decision-making and consent.

#### Participants' Safety and Withdrawal

In the unlikely event a participant suffers an adverse event as a result of participating in the research, the research will be suspended while investigation into the occurrence is considered and risk analysis is completed, inclusive of HREC advice being obtained and acted upon. Prospective participants will be advised that participation is voluntary and they may withdraw their interest and participation at any time. This information is documented in the PIS. If a participant withdraws after their contribution has been deidentified, the contribution or data will be unable to be extracted from the study.

#### Confidentiality

Participants will offer their voluntary consent to the research team using data to inform the research. Any information obtained in connection with the research that may identify participants will remain confidential.

#### Data Management

All data will remain confidential to the researcher and stored on a password-protected computer, and in the University of South Australia’s Research Data Storage and University of South Australia server. Access to data will be limited to the research team. Expert Advisory Group data may potentially be individually identifiable through voice recognition if recordings of Zoom contributions are made. These recordings may be required to ensure accurate documentation of participant contributions in a busy meeting setting. The researcher will undertake to deidentify this data at the point of transcription.

## Results

This research will identify an agreed set of core attributes required to develop and support successful disciplinary nurse leaders in academe.

## Discussion

### Anticipated Findings

It is anticipated that the findings of this study will inform the design and delivery of postgraduate nurse leadership programs and assist senior nursing faculty in HE to support the development of the next generation of academic nurse leaders. The findings will contribute to recruitment strategy, including succession planning and job description development. Upon completion of this research it is anticipated that the data sets and findings will be transferrable to other disciplines within HE to aid in future-proofing discipline-based expertise and leadership in the context of academic restructure.

### Limitations

The scoping review process will align to the PRISMA-ScR, and the selection of studies will be guided by a protocol reviewed by the research team with expertise in conducting scoping reviews and prospectively published on the Open Science Framework [19]. While the researchers aim to ensure the rigor of the scoping review design is upheld, potential limitations include relevant articles published in languages other than English that may be missed, the exclusion of articles that focus on clinical leadership, and characterization and interpretation bias. A data extraction tool will be used to ensure that the selected studies are independently mapped to the research questions by two of the researchers with conflicts reviewed by the third researcher [18].

The Delphi method has been adopted due to the ability to refine the thoughts and ideas of a large cohort of experts to form a consensus view; however, the limitations of this method include timeliness and the potential complexities of conducting multiple rounds to achieve agreement among participants [22,33]. The validity of the Delphi studies can be called into question due to the varied definitions of “consensus” [33]. While agreement of ≥80% is deemed sufficient to validate content, the researchers have set an agreement of ≥90% to ensure a high level of validity and increase the rigor of this study [22]. The researchers will use e-surveys to improve accessibility for participants in an effort to increase participation in and improve the validity of the study [33]. The use of e-surveys also reduces the likelihood of bias developing through participants who may seek to demonstrate the same view as the majority [33].

### Conclusions

The development of a set of agreed core attributes will assist current disciplinary leads to support early career trajectory identification and fast-tracking of the next generation of nursing disciplinary leaders in Australian and New Zealand HE institutions. It is anticipated that the findings of this research will inform postgraduate nurse leadership programs and assist senior nursing faculty in HE to effectively implement recruitment strategies, including succession planning and job description development. Ensuring the next generation of nursing disciplinary leaders hold or are supported to grow the qualities, behaviors, and characteristics that will empower them to succeed will assist in future-proofing discipline-based expertise and leadership in the context of academic restructure.

## Abbreviations

CDNM: Council of Deans of Nursing and Midwifery
HE: higher education
HREC: Human Research Ethics Committee
PIS: participant information sheet
PRISMA-ScR: Preferred Reporting Items for Systematic Reviews and Meta-Analysis Extension for Scoping Reviews
RN: registered nurse

## Data Availability

Data sharing is not applicable to this article as it outlines a prospective research protocol and therefore at this stage no data sets have been generated.
